# Spectrometer-Free Graphene Plasmonics Based Refractive Index Sensor

**DOI:** 10.3390/s20082347

**Published:** 2020-04-20

**Authors:** Li Zhang, Mohamed Farhat, Khaled Nabil Salama

**Affiliations:** Sensors Lab, Advanced Membranes and Porous Materials Center, Computer, Electrical and Mathematical Science and Engineering Division, King Abdullah University of Science and Technology (KAUST), Thuwal 23955-6900, Saudi Arabia; mohamed.farhat@kaust.edu.sa (M.F.); khaled.salama@kaust.edu.sa (K.N.S.)

**Keywords:** spectrometer-free sensing, graphene plasmonics, optical refractive index sensor

## Abstract

We propose a spectrometer-free refractive index sensor based on a graphene plasmonic structure. The spectrometer-free feature of the device is realized thanks to the dynamic tunability of graphene’s chemical potential, through electrostatic biasing. The proposed sensor exhibits a 1566 nm/RIU sensitivity, a 250.6 RIU^−1^ figure of merit in the optical mode of operation and a 713.2 meV/RIU sensitivity, a 246.8 RIU^−1^ figure of merit in the electrical mode of operation. This performance outlines the optimized operation of this spectrometer-free sensor that simplifies its design and can bring terahertz sensing one step closer to its practical realization, with promising applications in biosensing and/or gas sensing.

## 1. Introduction

Optical refractive index (RI) sensors are widely used due to their high sensitivity. Different structures for optical RI sensors have been developed over the past years, such as sensors based on optical fibers [1] and optical resonators [2,3,4,5]. Among these sensing devices, a group is based on the physical phenomenon of surface plasmon resonance (SPR). This type of biosensors is particularly used nowadays because it has proven its ability to provide a fast response in real time, without the use of markers. This stems from the singular property of surface plasmon polaritons (SPPs), to guide light along a metal/dielectric interface, and this precisely depends on the electromagnetic properties of the dielectric. More specifically, SPPs are surface waves whose characteristics are very sensitive to the dielectric medium, which is in contact with the surface of the metal. A small variation of the refractive index of this medium causes a change in the so-called resonance conditions. By measuring this change, it is then possible to detect, in real time, the presence of chemical and/or biochemical species in the vicinity of the metal surface and thus to achieve very sensitive sensors, for example, for quantitative analyzes of biomolecular reactions.

Several of these sensors involve more than one optical phenomenon to improve the capabilities (such as sensitivity, figure of merit, etc.) For instance, the sensor proposed in [5,6] combines whispering-gallery mode resonator and localized SPR (LSPR) mode, which leads to extremely high sensitivity. In [6], the proposed sensor can even realize single ion/molecule detection. For now, research groups have developed several different types of RI sensors with outstanding performance. However, most of these optical sensors require spectrometers, interferometers, prisms, or some other complex optical components. These components are usually bulky and thus not suitable for integration [7]. Therefore, novel designs that can discard these bulky elements have to be sought after, using spectrometer-free conceptions [7,8]. In [7] the spectrometer-free design is realized by measure of the transmission intensity change, while in [8], the sensor produces different reflection images as responses to the RI change. However, in [7] the measured optical intensity is usually more sensitive to noise from the source and detector than to the resonance peak. In [8], to get and analyze the reflection image, a CCD array and post-image processing are required which also increases the complexity of the system. Generally speaking, the key to the spectrometer-free design consists in finding another response to the RI change.

In our study, we propose a spectrometer-free design using graphene SPR-based structures. In fact, since the first isolation of graphene [9,10], this two-dimensional (2D) material has attracted an everlasting enthusiasm in the scientific community due to its unprecedented properties. Among these works, many have shown the importance of graphene as ideal plasmonic material [11,12,13] with excellent performance (e.g., perfect absorbers based on graphene [14,15,16,17] and high quality factor and sensitive graphene metamaterials [18]). Hence, different promising applications of graphene plasmonic structures have been put forward [19,20,21,22]. Sensing is one of these important uses of graphene, and different sensors based on this 2D material have been realized in the past few years [23,24,25]. Nevertheless, it should be emphasized that these graphene-based plasmonic sensors require complex optical components, to conveniently operate. In our work, we exploit the tunability of the graphene electrical response to realize a spectrometer-free terahertz sensor, using the electrical response of graphene sheets for molecular sensing. For instance, in [26] for example, the authors realized a spectrometer-free molecular sensing by studying the absorption of graphene sheets under different electrostatic biased chemical potentials. Under each chemical potential, there is a corresponding absorption peak in the frequency domain. By sweeping the chemical potential, the characteristic absorption peak from the analyte molecule is acquired. Finally, the corresponding resonance peak in the frequency domain may be resolved. However, the focus is more on the theoretical illustration of the concept. Here, we design an efficient sensor, adopting a somehow similar idea, using the electrical response to resolve the RI of the analyte medium. For instance, the conductivity of a graphene sheet is directly related to its chemical potential, which can be tuned using different mechanisms, e.g., electrostatic bias, chemical doping, and magnetic bias [27]. Since the graphene electrical property and the RI of the analyte (surrounding) medium are linked by the SPR mode, any change in the RI can be mapped into a change of the graphene chemical potential (or a bias voltage, in case electrostatic bias is used). Hence, a flexible realization of a spectrometer-free design may be obtained with different chemical potential tuning mechanisms. Moreover, we take into account the following considerations: Among these tuning mechanisms, chemical doping is a permanent tuning method by which we cannot continuously and reciprocally tune the chemical potential of the graphene sheet. The electrostatic biasing scheme offers, on the other hand, a fast on-off switching of the graphene SPR, and a dynamical tuning of its excitation frequency by adjusting the applied electrical voltage. By switching it off, one recovers the original graphene (undoped) sheet. Meanwhile, compared with magnetic bias, electrostatic bias doesn’t require external equipment to generate magnetic fields (the graphene sheet can be itself a part of the biasing electrode.) Therefore, we choose electrostatic biasing as our default mechanism for tuning graphene’s chemical potential. 

In this work, we propose a practical spectrometer-free RI sensor design using the electrical tunability of graphene sheet. The performance of the sensor is studied both by analytical calculations and numerical simulations. The results show that this design is flexible, robust, and with better combined sensing characteristics than comparable sensors. The paper is organized as follows. In the Results section, the sensing mechanism and the design of the proposed sensor are illustrated. After that, the sensor’s performance for different chemical potentials is analyzed, including the performance-boosting methods. Then, the sensing mechanism is investigated in the optical mode of operation (working as a traditional optical RI sensor) and electrical mode of operations (working as a spectrometer-free sensor). In a sense, electrical or optical mode of operation refers to the mode of operation of the sensor, i.e., whether the frequency or the chemical potential of graphene are held constant. In the Discussion part, the effect on the sensitivity of the device by the change of the grating size are briefly discussed and the proposed sensor is compared with other related works and shown to outperform them in terms of the combined sensitivity and figure of merit. Last, the Conclusion gives a summary of the findings of this contribution.

## 2. Results

### 2.1. Sensing Mechanism and Sensor Design

The sensing mechanism of the proposed sensor in this work is based on graphene surface plasmon resonance (GSPR). Under the SPR, a strong electromagnetic field, highly confined around graphene leads to a high absorption peak. Thanks to the GSPR mode’s sensitivity to small changes of the surrounding optical properties, the proposed graphene sensor may sense the refractive index of the neighboring medium.

Consider a typical GSPR based structure, e.g., a thin layer of graphene located in the middle and covered by two dielectric materials from the top and the bottom (these materials can be different.) By assuming a simple plane wave solution form and applying boundary conditions, we can obtain the dispersion relation of this sandwiched structure [28] (See Appendix A.) Also, graphene may be treated as a current sheet with corresponding boundary conditions, when the dispersion relation is derived [29].

Assuming that the medium to be characterized is on top of the graphene layer, Equation (A1) (See Appendix A) can be generalized into the dispersion relation with the permittivity (or equivalently the refractive index) of the analyte medium $\epsilon_{1}$ ($n_{1}$) as a parameter:

(1) $$\omega =f(\beta ){|}_{\epsilon_{1}}.$$

In Equation (1), f is a function of β (the wavenumber along the propagation direction) with fixed $\epsilon_{1}$. Equation (1) summarizes the basic sensing mechanism of a simple GSPR sandwich structure, whereas, a change of the medium’s refractive index leads to different dispersion relations.

Generally, coupling techniques are required to overcome the wavenumber mismatch between incident electromagnetic (EM) wave and GSPR mode. In our study, we make use of gratings in order to realize the required coupling [28]. Owing to phase-matching, the grating adds an extra term to the propagation constant, i.e.,:

(2) $$\beta =k_{\mathrm{inc}}\sin(\theta )+n\frac{2\pi }{p}.$$

In Equation (2), $k_{\mathrm{inc}}$ is the incident wavenumber, θ is the angle of incidence, n is an integer number, and p is the period of the grating. In our study, a normally incident plane wave is used (i.e., θ=0). Once the period and integer number *n* are chosen, the propagation constant is fixed, which leads to a specific GSPR mode. With a fixed β, we can rewrite Equation (1) as:

(3) $$\omega =g(\epsilon_{1}){|}_{\beta =\beta_{0}}.$$

Equation (3) shows the direct relationship between the incident wave frequency ω and the sensed medium permittivity $\epsilon_{1}$, which is the basic sensing mechanism for this sensor to work in the optical mode of operation, that requires a spectrometer. 

Thanks to the tunability of graphene conductivity, one has extra free variables and parameters in the dispersion relation of the GSPR structure. In [13], the conductivity and permittivity of a graphene sheet are expressed, leading to the relation between quantities e.g., mobility, chemical potential, and permittivity (see Appendix A). Then, the relationship between graphene chemical potential ($\mu_{c}$) and its permittivity can be written as:

(4) $$\epsilon_{g}=h(\omega ,\mu_{c}).$$

Here h is a function of both ω and $\mu_{c}$. If we have a monochromatic incident plane wave, we can rewrite Equation (3) by using Equation (4) as:

(5) $$\mu_{c}=s(\epsilon_{1}){|}_{\beta =\beta_{0},\omega =\omega_{0}}.$$

In Equation (5), s is a function of $\epsilon_{1}$ with fixed β and ω. Equation (5) shows the possibility of spectrometer-free sensing, using the tunability of graphene through its chemical potential. In fact, $\mu_{c}$ can be tuned by different techniques, e.g., electrostatic biasing and chemical doping. If the former biasing is used (as done in this work) to tune the chemical potential, this sensor operates in the electrical mode of operation and leads to spectrometer-free sensing.

**Figure 1:**
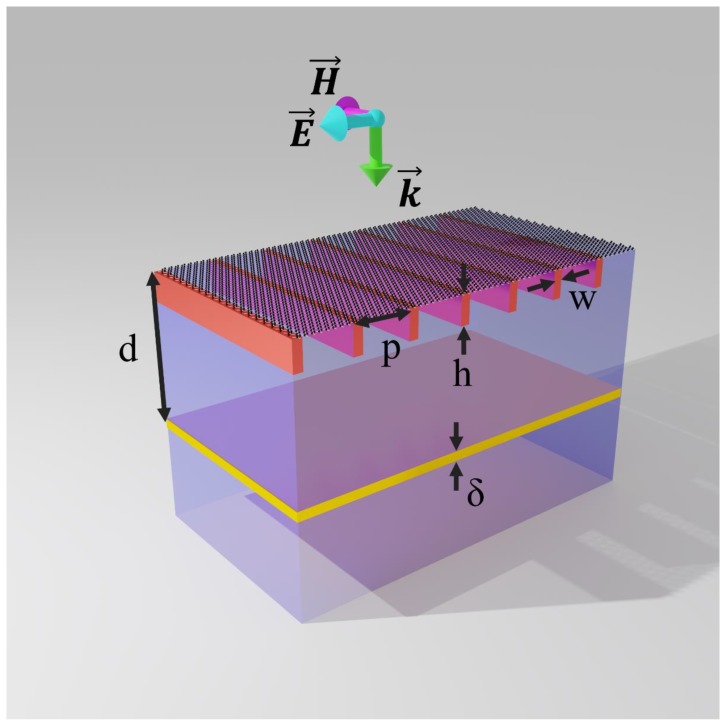
Cross-view of the proposed sensor structure, with its different geometrical parameters, and the normally incident plane wave excitation (polarization and wavevector.).

The graphene sensor structure is shown in Figure 1. This sensor consists of a grating coupled to a GSPR structure with a reflector, at the bottom. The basic structures of this sensor (responsible for the sensing mechanism) are the SPR sandwiched structures (the analyte medium on the top, a graphene layer, and a grating in the middle with a dielectric substrate, and the reflector on the bottom). The reflector located under the GSPR structure will reflect the transmitted energy back so that a higher absorption peak will appear which is easier to be detected by photodetectors. The geometrical parameters of this sensor are defined as, grating period p, grating height h, grating width w, and distance between the reflector and the graphene layer d. The thickness of the reflector will not affect the device performance significantly, as long as it is thick enough to reflect the transmitted energy back (the thickness δ in this study is chosen to be 50 nm), therefore, it can be designed flexibly according to the fabrication process capabilities. This device is designed to work for a normally-incident, transverse-magnetic (TM) polarized electromagnetic wave, as schematized in Figure 1. 

In this work, we first fix the grating period $p\approx 2\lambda_{\mathrm{GSPR}}=384.8\;\mathrm{nm}$ and GSPR mode propagation constant $\beta \approx 70k_{0}$ (for $\mu_{c}=700\;\mathrm{meV}$), which corresponds to a feasible surface plasmonic resonance mode with regards to the physical dimensions of the structures to be used. Furthermore, the grating width w and height h are set to 40 nm and 50 nm, respectively. A summary of the designed dimensions is shown in Table 1. The material properties (permittivity, mobility, etc.) of the designed devices are set with reasonable values from the common nanofabrication process. For the dielectric substrate, the relative permittivity is set to 3.5. As for graphene, mobility is set to 50,000 cm^2^/(V·s), which is a reasonable value for graphene with good quality [30] (see Section 3.3.) The metallic reflector is chosen to be made from gold with permittivity calculated using the Drude’s model [31].

### 2.2. Performance Analysis and Simulation Results

To quantitatively evaluate the performance of the sensor, sensitivity, figure of merit, limit of detection and specificity are commonly used. Since our sensor is a general RI sensor that does not involve molecular detection, specificity is not applicable in this study. 

In the optical mode of operation, we use the common definition of sensitivity, figure of merit (*FoM*) and detection limit (*DL*) [32] for optical RI sensor, i.e.,:

(6) $$\left\{ \begin{array}{l} S=\frac{\Delta \lambda (\mathrm{nm})}{\Delta n(\mathrm{RIU})},\\ FoM=\frac{S(\mathrm{nm}/\mathrm{RIU})}{FWHM(\mathrm{nm})},\\ DL=\frac{R(\mathrm{nm})}{S(\mathrm{nm}/\mathrm{RIU})}, \end{array}\right.$$

where the sensitivity S is defined as the ratio between the peak shift in wavelength and its corresponding refractive index change and the FoM is defined as the sensitivity over the peak full width at half maximum (FWHM). From the very definition of *FoM*, one can see that it is easier to distinguish between two resonance peaks under a certain RI change provided that the sensor has a higher *FoM*. Therefore, *FoM* is a critical characteristic related to the resolution of the sensor and a high *FoM* is desirable for enhanced sensitivity. Except from *FoM*, the resolution of the spectrometer and the noise parameters also determine the resolution of the sensor [32], which describe the ability to accurately determine the spectral shift. With the resolution of the sensor, we can define the detection limit of the sensor which equals the resolution divided by the sensitivity. 

As this graphene sensor has an additional spectrometer-free working mode (i.e., the electrical mode of operation), we use a similar approach to define the counterpart sensitivity, *FoM* and *DL* in that scenario, i.e.,:

(7) $$\left\{ \begin{array}{l} S=\frac{\Delta \mu_{c}(\mathrm{meV})}{\Delta n(\mathrm{RIU})},\\ FoM=\frac{S(\mathrm{meV}/\mathrm{RIU})}{FWHM(\mathrm{meV})},\\ DL=\frac{R(\mathrm{meV})}{S(\mathrm{meV}/\mathrm{RIU})}. \end{array}\right.$$

In this study, only calculations and simulations are done to examine the sensor performance. Hence, the noise level (including noise from the excitation laser, photodetector and auxiliary electric circuit) and spectral resolution (including the resolution of the tunability of excitation laser and the spectrometer) may not be acquired without an experimental implementation. Moreover, without loss of generality, only sensitivity and figure of merit will be calculated to evaluate the performance of the sensor.

After setting up the basic definitions, we can start by analyzing the sensitivity under different graphene chemical potentials by analytical means [solving Equations (3) and (5)] and finite-elements based simulations (using COMSOL Multiphysics^®^) with designed β and p. The sensitivities from both equations and simulations are obtained when the relative permittivity of the analyte medium is varied around the central value 3.5. Practically, in Equations (3) and (5) both g and s cannot be solutions expressed in a direct analytical formula. Therefore, during the solving process, the values of both sides of Equation (A1) are calculated with different sets of variables. By finding the minimum error between both sides, the relation between variables can be acquired numerically. 

**Figure 2:**
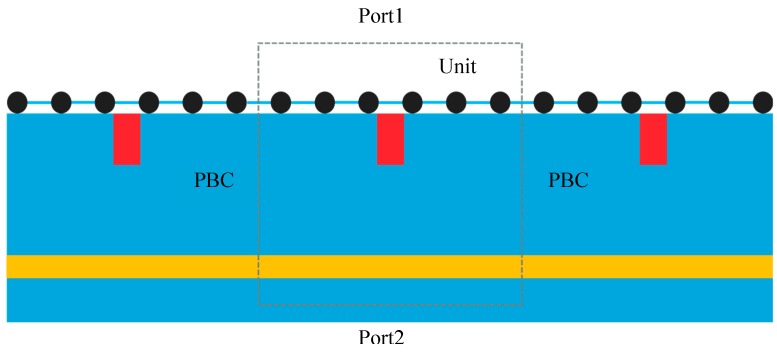
Simulation settings, showing the unit-cell and its boundary conditions.

For the simulations, we treat the whole sensor structure as a repeated sequence of unit-cells (shown in Figure 2). To characterize the whole structure, we make use of periodic boundary condition (PBC) at both sides of the unit-cell. In addition, the top and bottom boundaries are set as port 1 and port 2, respectively and the plane wave excitation is enforced at port 1. The scattering parameters (*S*-parameters) are calculated to evaluate the relationship between incident and reflected wave intensity. The simulations are performed both with reflector and without reflector, respectively. For the simulations with a reflector, a preliminary simulation is done in order to optimize the distance between graphene and the reflector so that we obtain the highest absorption peak.

**Figure 3:**
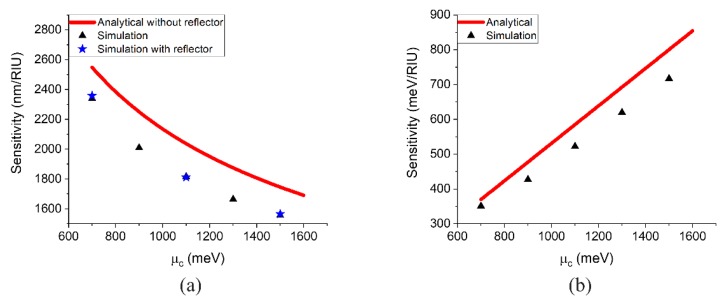
Simulated (triangles and stars) and analytically calculated (solid lines) sensitivity versus graphene chemical potential for (**a**) the optical mode of operation and (**b**) the electrical mode of operation.

As can be seen from Figure 3a, when $\mu_{c}$ is varied from 700 meV to 1600 meV, the calculated optical mode of operation sensitivity for the ideal GSPR structure decreases. Meanwhile, the simulated optical mode of operation sensitivity for the device structure (GSPR and grating) shows the same trend as the calculated one. However, the existence of gratings slightly changes the GSPR mode, which leads to the small discrepancy between simulations and analytical results by solving Equation (A1). Besides, the results with and without the metallic reflector are identically the same, which shows that the inclusion of the reflector will not evidently influence the sensitivity of the device. The sensitivity results for the electrical mode of operation are shown in Figure 3b. On the contrary, the sensitivity for the electrical mode of operation increases with increasing chemical potential. The simulation results also have the same trend as the calculated results with deviation due to the existence of gratings. In order to have perfect agreement between the simulation results and analytical results, one needs to add the effect of the grating, but this will make the problem insolvable analytically. However, the results of Figure 3 illustrate the sensing mechanism of the proposed device and give the trend of the sensitivity versus $\mu_{c}$.

**Figure 4:**
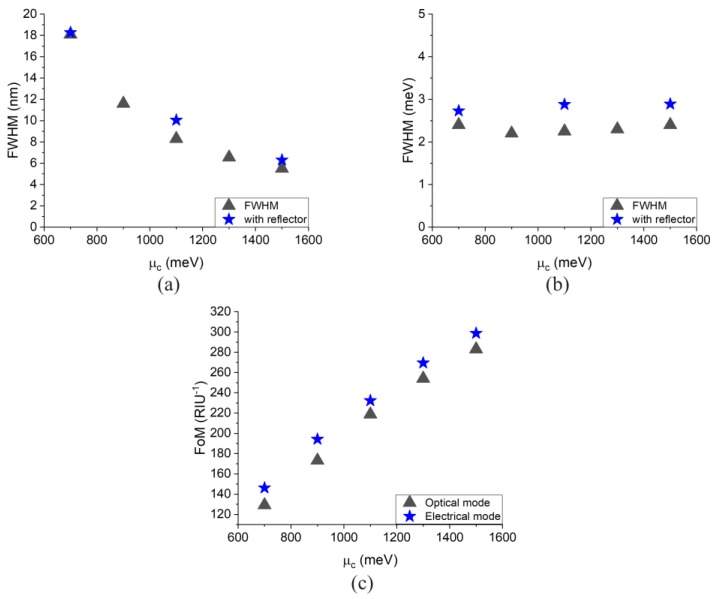
Simulated results versus graphene chemical potential for (**a**) *FWHM* in the optical mode of operation, (**b**) *FWHM* in the electrical mode of operation, and (**c**) *FoM* in both optical and electrical mode of operations.

Afterwards, all the simulated sensitivities and *FWHMs*, *FoMs* for both optical mode of operation and electrical mode of operation under different graphene chemical potentials are calculated and shown in Figure 4. For both modes, the *FoMs* increase linearly with the graphene’s chemical potential. Different from the sensitivities for both modes, the *FWHMs* and *FoMs* become slightly deteriorated due to the existence of the reflector.

The *FWHM* is also simulated versus different graphene chemical potentials. Figure 4a shows that the *FWHM* decrease as $\mu_{c}$ increases for both cases, with and without reflector. Different from the optical mode of operation, as can be seen from Figure 4b, the *FWHM* of the electrical mode of operation is almost independent of the change of $\mu_{c}$.

**Figure 5:**
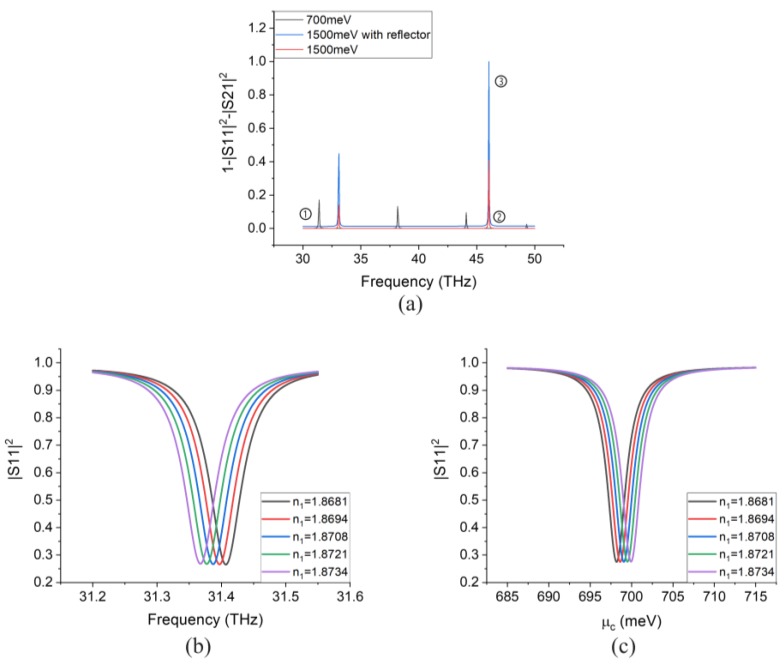
(**a**) Absorption spectrum for different scenarios. (**b**) Reflection coefficient ($S_{11}$) for the device with reflector working in the optical sensing mode with $\mu_{c}=700\;\mathrm{meV}$ for different permittivities. (**c**) Reflection coefficient for the device with reflector working in the electrical sensing mode with the same $\mu_{c}=700\;\mathrm{meV}$.

Figure 5a shows the absorption spectrum for different scenarios: for a structure with graphene’s chemical potential $\mu_{c}=700\;\mathrm{meV}$ and no reflector, the absorption peak corresponding to the design β is denoted by ①. For the case of $\mu_{c}=1500\;\mathrm{meV}$ and no reflector, the corresponding peak is ②. After including the reflector, the peak ② increases dramatically and transforms into peak ③, which almost reaches perfect-absorption [33]. Generally, by adding the gold reflector and simultaneously by increasing $\mu_{c}$, we can realize stronger absorption. The simulation results after adding the reflector to the device in the case of 700 meV chemical potential are shown in Figure 5b,c, for the optical and electrical mode of operations respectively. Since we add the reflector (transmission through port 2 is 0), the reflection equals the total incident power subtracted by the absorption, therefore, the reflector results in stronger absorption and converts the absorption peak to a reflection dip, which is easier to measure practically.

Summarizing all the observations above, a higher graphene’s chemical potential leads to better *FoMs* for both optical mode of operation and electrical mode of operation and better sensitivity for the electrical mode of operation, while decreasing the sensitivity for the optical mode of operation. (i.e., for the optical mode of operation, the choice of chemical potential will lead to a trade-off between sensitivity and *FoM*.) The inclusion of the reflector will not affect the sensitivity and *FoM* alike, but it helps to realize stronger absorption peak (or weak reflection dip), which may reduce the constraint on sensitivity of the detecting devices such as photodiodes. Therefore, for the final design, graphene chemical potential is chosen to be 1500 meV with a gold reflector on the bottom structure. 

**Figure 6:**
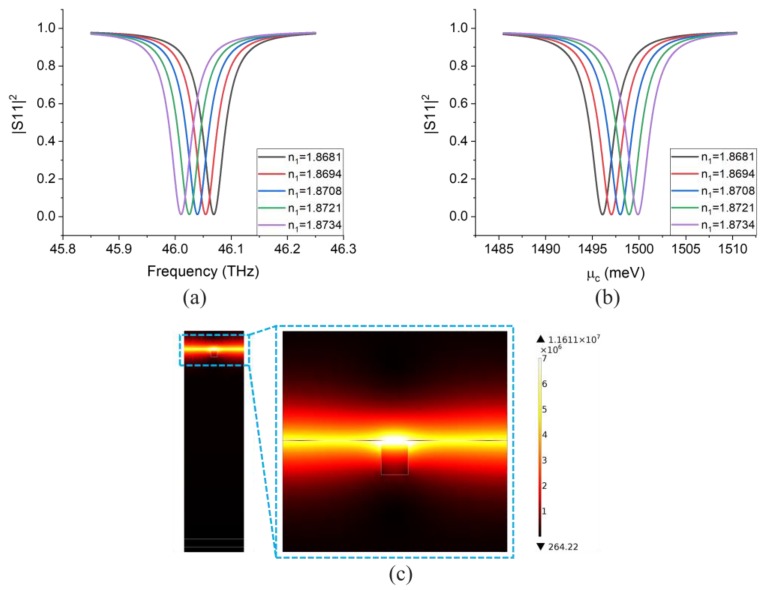
(**a**) Reflection coefficient for the sensor (with reflector) working in the optical mode of operation with $\mu_{c}=1500\;\mathrm{meV}$. (**b**) Reflection coefficient for the same device working in the electrical mode with the same chemical potential. (**c**) Simulated electric field intensity at GSPR peak for $\epsilon_{1}=3.5$ ($n_{1}=1.87$).

The simulation results of the chosen design, based on the abovementioned assumptions, are shown in Figure 6a,b where the reflection coefficient of the optical mode of operation and the electrical mode of operation are given, respectively. It is clear that the minimum reflection coefficients for both cases are nearly zero (perfect-absorption), which are even better than the results of Figure 5b,c. The perfect-absorption is realized by optimizing the distance between the graphene and reflector (here d=1244 nm). Next, we can calculate the sensitivity and *FoM* from Figure 6a,b. The calculated sensitivity and *FoM* for the optical mode of operation are 1566.03 nm/RIU and 250.6 RIU^−1^, respectively. For the electrical mode of operation, the sensitivity and *FoM* are 713.21 meV/RIU and 246.8 RIU^−1^, respectively. Figure 6c plots the electric field intensity of the device at the resonance peak, shown for one period of the grating (there are four wave nodes corresponding to two wavelengths of the GSPR mode). When the device is operating at the resonance peak, the fields are, as expected, highly confined within a narrow area around the graphene layer due to the property of GSPR. 

**Figure 7:**
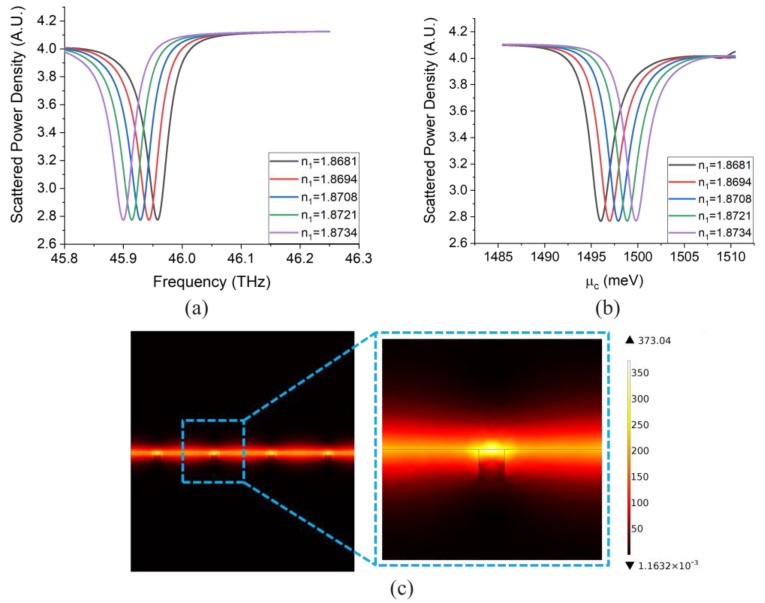
(**a**) SCS for a device with 40 unit-cells and reflector working in the optical mode of operation with $\mu_{c}=1500\;\mathrm{meV}$. (**b**) Scattered power density for the same device working in the electrical mode of operation with the same chemical potential. (**c**) Simulated electric field intensity at the GSPR peak when $\epsilon_{1}=3.5$ ($n_{1}=1.87$).

To further investigate the feasibility of this device for a realistic configuration, an additional simulation is performed with a finite device structure (finite number of unit-cells of the grating). In this scenario, the device consists of only 40 units. A background normally-incident plane wave impinges on the device and the total scattered power density is calculated (by integrating the Poynting vector in the far-field over a closed surface enclosing the structure). The simulation results for the scattering cross-section (SCS) are shown in Figure 7.

From Figure 7a, it can be observed that with the finite structure, the peaks in the optical mode operation are slightly redshifted in comparison to those of Figure 6a (i.e., for the infinite periodic structure), while the peaks in the electrical mode of operation shown in Figure 7b are almost the same as those of Figure 6b. The sensitivities and *FoMs* calculated from Figure 7a,b are 1561.5 nm/RIU, 269.2 RIU^−1^, and 713.4 meV/RIU, 250.5 RIU^−1^, respectively, which are very close to those calculated from Figure 6a,b. Additionally, from Figure 7c, it can be clearly seen that the finite structure device has the same optical mode as the infinite periodic one. Generally, it can be concluded that the device with finite periodic structure operates in the same way as the infinite periodic structure and possesses the same performance (in terms of sensitivity and *FoM*.)

**Figure 8:**
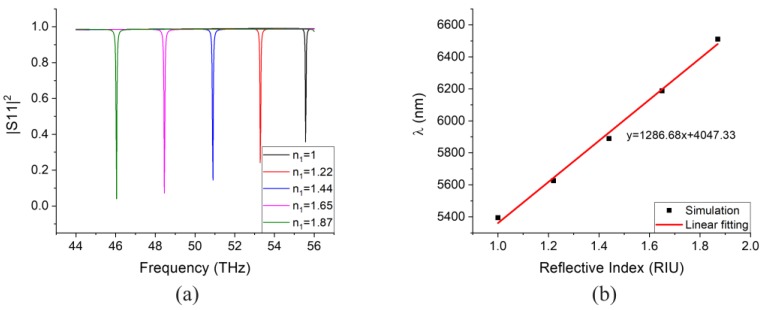
(**a**) Reflection coefficient for the device with infinite periodic structure and reflector working in the optical mode of operation with $\mu_{c}=1500\;\mathrm{meV}$ under large medium refractive index change. (**b**) Peak wavelength versus corresponding medium refractive index and its linear fitting.

In order to investigate the sensor’s performance under significant RI changes, an additional simulation is performed with large analyte medium refractive index change. Figure 8a depicts the peak-shift for different medium RIs. As can be seen, when the RI increases, the resonance peak redshifts (i.e., towards lower frequencies). Further, when the RI has a value that is very far from the original design value (*n* = 1.87), the absorption strength drops, because the reflector position is fixed and optimized to the original design. In other words, we can optimize the reflector distance according to different sensing cases. Figure 8b shows the relation between the peak wavelength and the corresponding medium refractive index. This sensor can retain a relative linear performance spanning different RIs. The sensitivity under large RI change is 1286.68 nm/RIU according to the linear fitting, shown in Figure 8b.

## 3. Discussion

### 3.1. Geometrical Optimization

In the previous sections, we analyzed the role of graphene’s chemical potential and reflector position on the GSPR sensing mechanism. The objective of the following discussion is to understand the influence of the geometric parameters of the grating on the sensor’s performance.

To investigate the influence of the grating’s width, several simulations with varying widths are performed, as before. In each simulation, the distance between the reflector and the graphene layer is optimized to obtain the highest resonance peak. As can be seen from Table 2, the narrower the grating is, the better the performance of the GSPR sensor. This improvement is more obvious for the *FWHM*, that is halved (from 6.3 nm to 3 nm) when the grating’s width is almost halved (from 40 nm to 18 nm), which is a huge improvement. However, the same geometrical change leads to an improvement of only around 5% of the sensitivity (see Table 2). It should be also emphasized that shrinking the width of the grating to the nanometer scale, puts a high constraint on currently available nanofabrication technology. Therefore, a trade-off between the performance of the sensor and the fabrication complexity has to be met. 

In the same vein, we investigate the influence of the grating’s height and the corresponding optimized reflector distance on the spectrometer-free GSPR sensor. The results are summarized in Table 3. When the height is divided by two, the performance in terms of *FWHM* and sensitivity is almost unaltered. This can be explained by the fact that the electromagnetic field is highly confined within a short distance around the graphene (plasmonic) layer, due to the GSPR effect. Thus, the electromagnetic fields do not sense, in a certain way, the thickness of the grating, if these are larger than the confinement distance (few nanometers, in this case). Therefore, it can be concluded that the performance of the sensor is insensitive to the grating height, and this parameter can be flexibly designed, according to the nanofabrication process.

### 3.2. Performance Comparison

To demonstrate the advantage of this sensor, we make a comparison of the device’s performance with other state of the art recent works. Since the novelty of this sensor comes from its electrical mode of operation, and due to the lack of sensors to compare with, in this specific regime, and as our sensor operates in both modes, this comparison is done in the optical mode of operation, without loss of generality. For instance, in [34], a terminal optical fiber SPR sensor is proposed. In this design, the medium that supports the SPR mode is gold with carbon nanotube and Pt nanoparticles. This sensor can achieve a high sensitivity of 5923 nm/RIU but possesses a relatively low *FoM*, while in Ref. [35], the opposite situation occurs. This single layer guided-mode resonant sensor in [35] features with a high *FoM* of 690 RIU^−1^. In [36], the performance of the proposed sensor is not outstanding, however, it is insensitive to the incident wave polarization. In [37,38,39], the sensors come with good sensitivity but relatively low *FoM*.

**Figure 9:**
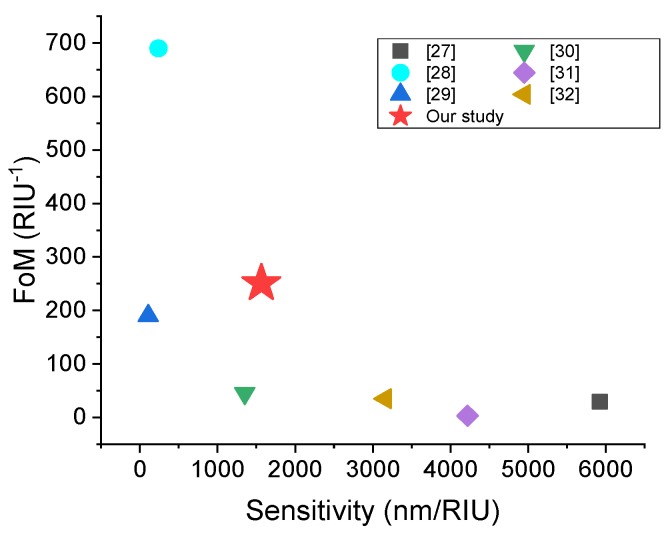
Performance comparison of *FoM* and sensitivity of the sensor proposed in this study to similar other sensors. The star denotes the current study.

The performance data for the considered sensors are plot in Figure 9. Higher sensitivity means larger change under certain RI variation, which releases the requirement of the spectrometer. Higher *FoMs* result in sharper peak and less overlap between peaks under small RI change, which relaxes the demand on the distinction of the peaks. Usually, the peak distinction process is done by peak-fitting or other algorithms that require data processing. As can be seen, the sensor proposed in our study has a more balanced operation (good sensitivity and *FoM*, simultaneously), which relaxes both the requirements of the spectrometer and peak distinction process. This comparison illustrates that our sensor has a comparable/better performance even in the traditional optical mode of operation, in addition to its operation in the electrical (spectrometer-free) mode.

### 3.3. Mobility, Gating, and Advantages of the Sensor

A mobility of 50,000 cm^2^/(V·s) was assumed in this work [27,40,41,42], however, if graphene features a relatively lower mobility (lower quality), the SPR will be degraded and may exhibit a broader reflection dip, which leads to a lower *FoM*. Also, the size of the gratings has an effect on the device performance. Since the SPR mode is highly confined around the graphene layer, the height of the grating will not have an obvious effect on the device operation. However, the width of the grating will influence the SPR mode. Therefore, the grating width used in the simulations is well traded-off between device performance and nanofabrication technology limits.

Furthermore, an important practical aspect of the proposed sensing method is the control of graphene’s Fermi level (or chemical potential). One possible way may be to use the metal-reflector as a second electrode and that this may result in high voltages. However, we are inspired by the experimental setup demonstrated in [21] where similar devices were investigated with voltages up to few hundred volts, used to tune the chemical potential of graphene. We believe that such setup can be feasible with our device. However, it is also possible to use different configurations, i.e., using extraordinary optical transmission electrodes (EOT) as was suggested for example in [21,43]. These electrodes can be made transparent in the frequency domain of interest and conserve their conductivity.

Additionally, in practical applications, the laser source used to excite our device has a finite linewidth of its power spectrum rather than an ideal monochromatic spectrum. Therefore, we qualitatively examine the device performance under the excitation of a finite linewidth source (See Appendix B). The calculation reveals that the effect by a finite linewidth of the source on the sensitivity of the device is negligible. However, the figure of merit will be degraded by the broader band of the source.

To sum-up, the proposed sensor has performance that overcomes classical (optical) biosensors, and its advantages over the state-of-the-art sensors are summarized below:

(1) *Spectrometer-free*: The design does not require the use of a spectrometer, which relaxes the design and reduces its complexity, via the electrical mode of operation.
(2) *Higher performance*: The GSPR sensor possesses simultaneously a high *FoM* and sensitivity. This equips the proposed design with a higher precision in detection, in the terahertz range.
(3) *Geometry robustness*: The operation is insensitive to the height of the grating, which gives more flexibility in the design and robustness regarding the imperfections in nanofabrication.
(4) *Dynamic tunability*: The spectral location of the EM absorption peak can be tuned by changing $\mu_{c}$. This shows that the same device can be optimized for different frequency ranges (with reconfigurability, by use of electrostatic biasing).

## 4. Conclusions

In this paper, a spectrometer-free sensing design is proposed and characterized through optical numerical simulations. This sensor consists of a GSPR structure with gratings for coupling to incident EM waves and a metallic reflector to boost the performance to have a higher absorption peak. The sensor can operate in both optical mode of operation and electrical mode of operation. The device’s performance for different graphene chemical potentials is investigated. These simulations assume a relative permittivity of the analyte medium varying around 3.5 and a chemical potential $\mu_{c}=1500\;\mathrm{meV}$ is chosen for the final design. In the optical mode of operation, a sensitivity and *FoM* of 1566.03 nm/RIU and 250.6 RIU^−1^, respectively were demonstrated. On the other hand, for the electrical mode of operation, these were 720.75 meV/RIU and 287.2 RIU^−1^. In addition, a finite-size device (consisting of only 40 unit-cells) is also characterized and it is subsequently shown that it has almost the same performance as the infinitely periodic structure. The robustness of the sensor with respect to geometric variations was further investigated, and the results show that this device can be further optimized with a smaller width but at the expense of the complexity of the fabrication process. Last but not least, a comparison of the proposed sensor’s capabilities with similar works is drawn, and it is demonstrated numerically that the combination of the four advantages (mentioned in the discussion section) significantly increases the sensitivity and *FoM* of the proposed device in comparison with the listed designs.

The new proposed device made of graphene is an important step for sensing for several reasons. On the one hand, this simple technique shows that it is possible to perform a complex analysis on a single device, without the need of a spectrometer, whereas it usually requires many different processes. On the other hand, it shows the unprecedented potential of graphene in sensing, which may lead to several possible applications in biosensing, e.g., for biomolecules, polymers, and many other substances.

## Figures and Tables

**Table 1:** Designed sensor geometry (See Figure 1).

Geometry Dimensions	Value (nm)
*p*	384.8
*w*	40
*h*	50
δ	50
*d*	1244.4

**Table 2:** Simulated sensitivity and *FWHM* for different grating widths (see Figure 1).

Grating Width (nm)	*FWHM* (nm)	Sensitivity (nm/RIU)
40	6.3	1566.03
26	4.5	1603.8
18	3	1641.5

**Table 3:** Simulated sensitivity and *FWHM* for different grating’s heights.

Grating Height (nm)	*FWHM* (nm)	Sensitivity (nm/RIU)
50	6.3	1566.03
25	6.25	1566.04

**Figure A1:**
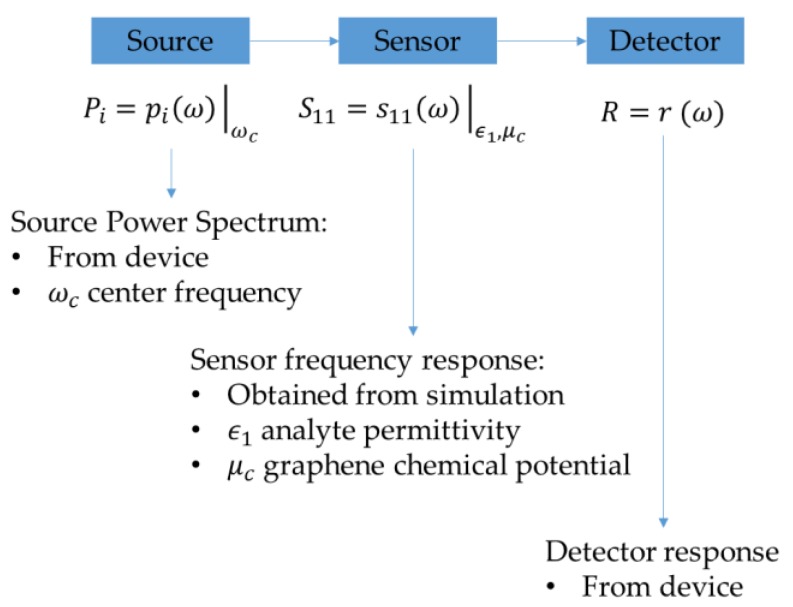
Systematic composition of the sensing setup in frequency domain.

## Appendix A. Dispersion Relation and Graphene Conductivity

In [28], the dispersion relation of a three-layer sandwich structure is derived:

(A1) $$\left\{ \begin{array}{ll} e^{-2tk_{g}}=\frac{\left(\frac{k_{g}}{\epsilon_{g}}+\frac{k_{2}}{\epsilon_{2}})(\frac{k_{g}}{\epsilon_{g}}+\frac{k_{1}}{\epsilon_{1}}\right)}{\left(\frac{k_{g}}{\epsilon_{g}}-\frac{k_{2}}{\epsilon_{2}})(\frac{k_{g}}{\epsilon_{g}}-\frac{k_{1}}{\epsilon_{1}}\right)} & \left(a\right)\\ k_{i}^{2}=\beta^{2}-k_{0}^{2}\epsilon_{i}\;,\;i=g,1,2 & \left(b\right) \end{array}\right.$$

In Equation (A1), t is the thickness of the graphene layer, $k_{1}$, $k_{g}$, $k_{2}$ are the wavenumbers in the perpendicular direction for top dielectric, graphene layer, and bottom dielectric, respectively. $\epsilon_{i}$ ($\sqrt{n_{i}}$) is the permittivity (refractive index) in the corresponding region, β is the propagation wavenumber parallel to the graphene layer, and $k_{0}$ is the free-space wavenumber.

Then, the conductivity and permittivity of graphene can be expressed as [13]:

(A2) $$\begin{array}{l} \sigma_{\mathrm{intra}}=\frac{ie_{0}{}^{2}k_{B}T}{\pi \hbar^{2}(\omega +i2\Gamma )}[\frac{\mu_{c}}{k_{B}T}+2\ln(e^{-\frac{\mu_{c}}{k_{B}T}}+1)],\\ \sigma_{\mathrm{inter}}\approx \frac{ie_{0}{}^{2}}{4\pi \hbar }\ln[\frac{2|\mu_{c}|-(\omega +i2\Gamma )\hbar }{2|\mu_{c}|+(\omega +i2\Gamma )\hbar }]\;,k_{B}T<<|\mu_{c}|,\\ \Gamma =\frac{e_{0}{\left(\frac{c}{300}\right)}^{2}}{2\mu \mu_{c}},\\ \sigma =\sigma_{\mathrm{intra}}+\sigma_{\mathrm{inter}},\\ \epsilon_{g}=1+i\frac{\sigma }{\epsilon_{0}\omega t}. \end{array}$$

In Equation (A2), $\sigma_{\mathrm{intra}}$, $\sigma_{\mathrm{inter}}$ and σ (is −2.81×10^−7^+6.06×10^−4^i S/m under our working frequency of 46.05 THz with $\mu_{c}=1500\;\mathrm{meV}$) represent intra-band conductivity, inter-band conductivity, and total conductivity of graphene, respectively. $e_{0}$ is the elementary electronic charge, $\mu_{c}$ is the chemical potential of graphene, μ is the electron mobility of graphene, $\epsilon_{0}$ and $\epsilon_{g}$ (−2.36×10^2^ − 1.10×10^−1^ i under our working frequency of 46.05 THz with $\mu_{c}=1500\;\mathrm{meV}$) are the vacuum permittivity and graphene relative permittivity, respectively. With both Equations (A1) and (A2), the GSPR properties can be analyzed. From a qualitative point of view, some simple conclusions can be made about how the conductivity and effective permittivity of graphene sheet may affect the sensor performance. If we keep the same resonance mode (i.e., fixed β defined by the gratings) and consider that β is much larger than $k_{0}$, we can conclude that $k_{1}$, $k_{g}$, $k_{2}$ remain almost unchanged, then the effective permittivity of the graphene sheet $\epsilon_{g}$ has to remain unchanged. Therefore, a higher conductivity will lead to a higher resonant frequency. Also, a higher conductivity will lead to a resonance with less damping. (i.e., smaller *FWHM*).

## Appendix B. Device Performance Under Finite Band Excitation

The proposed device operates under moderate optical intensity, therefore, it can be treated as a linear system (nonlinear effects can be neglected). Considering the whole sensing system, it consists of the excitation source, the sensing device, and the detector (As shown in Figure A1). Therefore, the overall response (optical power $P_{o}$ sensed by the detector) that we may read from the detector is the integration of the product of the power frequency response of each individual component in the system over the entire frequency domain, i.e.,:

(A3) $$P_{o}{|}_{\omega_{c},\epsilon_{1},\mu_{c}}=\int_{0}^{\infty }p_{i}{|}_{\omega_{c}}S_{11}{|}_{\epsilon_{1},\mu_{c}}rd\omega .$$

If we further consider a finite spectrum of the source, the integration domain is:

(A4) $$P_{o}{|}_{\omega_{c},\epsilon_{1},\mu_{c}}=\int_{\omega_{l}}^{\omega_{h}}p_{i}{|}_{\omega_{c}}S_{11}{|}_{\epsilon_{1},\mu_{c}}rd\omega .$$

In Equation (A4), $\omega_{h}$, $\omega_{l}$ are limiting frequencies of the source power spectrum. For the sake of simplicity, we assume that the source power spectrum is a normal distribution function (with standard deviation equals to 0.02 THz and 0.01 THz [44,45]) and an ideal detector (r=1). With $S_{11}$ from the simulation, $P_{o}$ can be calculated using Equation (A4), then the device performance with finite band excitation can be acquired:

As can be seen from Table A1, the finite band of the source will have a negligible effect on the sensitivity but an effect on the figure of merit. However, as the quality of laser source being improved, it’s possible to have comparable performance as the monochromatic case.

**Table A1:** Calculated sensitivity and FoM under the finite band excitation.

Mode of Operation	Sigma (THz)	FoM (RIU^−1^)	Sensitivity
Optical	0.02	149.7	1557.1 nm/RIU
	0.01	204.8	1556.6 nm/RIU
Electrical	0.02	148.9	714.5 meV/RIU
	0.01	204.1	714.4 meV/RIU
